# School Children

**Published:** 1858-01

**Authors:** 


### SCHOOL CHILDREN.

MANY a child, the light of the house today, will have been laid in the grave before the winter is ended, by inattention as to heat and cold, inducing pleurisies, inflammation of the lungs, colds, croups, and other dangerous maladies.

Teachers should be spoken to about allowing the children to sit with the back near a stove, or register, or window, or in any position where the child is exposed to a draft of air, or to overheat.

The children should not be allowed to come directly to a fire, or stove, on entering the school room.

In addition, they should be detained in an outer room fifteen or twenty degrees colder, for a few minutes after the school is dismissed, and then have their gloves put on, and a vail put over the face and fastened, so as not to be blown aside. The colder the weather, and the higher the wind, the more necessary are these precautions, not only in leaving the school-room, but on leaving home.

The grateful relief which is experienced when facing a fierce cold wind, on putting a silk handkerchief over the face, will surprise any one who tries it.

All india-rubber shoes or garments should be removed the moment on coming in-doors.

Children should be instructed to run with the mouth shut for the first block or two after getting out of doors in cold weather.

